# *BRAF* mutations and phosphorylation status of mitogen-activated protein kinases in the development of flat and depressed-type colorectal neoplasias

**DOI:** 10.1038/sj.bjc.6602911

**Published:** 2005-12-13

**Authors:** K Konishi, M Takimoto, K Kaneko, R Makino, Y Hirayama, H Nozawa, T Kurahashi, Y Kumekawa, T Yamamoto, H Ito, N Yoshikawa, M Kusano, K Nakayama, B J Rembacken, H Ota, M Imawari

**Affiliations:** 1 Second Department of Internal Medicine, Showa University School of Medicine, 1-5-8 Hatanodai, Shinagawa-ku, Tokyo 142-8666, Japan; 2 Second Department of Pathology, Showa University School of Medicine, Tokyo, Japan; 3 Clinical Research Laboratory, Showa University School of Medicine, Tokyo, Japan; 4 Endoscopy center, Showa University Hospital, Tokyo, Japan; 5 Second Department of Surgery, Showa University School of Medicine, Tokyo, Japan; 6 Department of Obstetrics and Gynecology, Shimane Medical University, Izumo, Japan; 7 Centre for Digestive Disease, The General Infirmary, Leeds, UK

**Keywords:** depressed neoplasia, flat adenoma*BRAF* mutation, colorectal carcinoma, MAPK pathways, colorectal polyp

## Abstract

Although some molecular differences between flat-depressed neoplasias (FDNs) and protruding neoplasias (PNs) have been reported, it is uncertain if the *BRAF* mutations or the status of phosphorylated mitogen-activated protein kinase (p-MAPK) are different between theses two groups. We evaluated the incidence of *BRAF* and *KRAS* mutations, high-frequency microsatellite instability (MSI-H), and the immunohistochemical status of p-MAPK in the nonserrated neoplasias (46 FDNs and 57 PNs). *BRAF* mutations were detected in four FDNs (9%) and none of PNs (*P*=0.0369 by Fisher's exact test). *KRAS* mutations were observed in none of FDNs and in 14 PNs (25%; *P*=0.0002 by Fisher's exact test). MSI-H was detected in seven out of 44 FDNs (16%) and in one out of 52 of PNs (2%) (*P*=0.022 by Fisher's exact test). Type B and C immunostaining for p-MAPK was observed in 34 out of 46 FDNs (72%), compared with 24 out of 55 PNs (44%; *P*=0.0022 by *χ*^2^ test). There was no significant difference in the type B and C immunostaining of p-MAPK between FDNs with and without *BRAF* mutations. *BRAF* and *KRAS* mutations are mutually exclusive in the morphological characteristics of colorectal nonserrated neoplasia. Abnormal accumulation of p-MAPK protein is more likely to be implicated in the tumorigenesis of FDNs than of PNs. However, this abnormality in FDNs might occur via the genetic alteration other than *BRAF* or *KRAS* mutation.

The adenoma–carcinoma sequence is well accepted as a major pathway for the development of colorectal cancers (CRCs) (Morson, 1968). Most CRCs are thus believed to arise from pre-existing adenomatous polyps. Most of these benign precursor lesions are of polypoid, protruding origin (Muto *et al*, 1975); however, many investigators have reported flat and depressed neoplasias (FDNs) as a new type of precursors of CRC and propose that these tumours develop through a *de novo* pathway, as they are not associated with adenoma components (Bedenne *et al*, 1992; Iishi *et al*, 1992; Kudo, 1993; Minamoto *et al*, 1994b; Konishi *et al*, 1999; Rembacken *et al*, 2000; Saitoh *et al*, 2001). Flat-depressed neoplasias are characterised by a higher potential of malignancy than protruding neoplasias (PNs) (Kudo, 1993). Small nonpolypoid cancers have particularly greater aggressiveness than polypoid cancers of equivalent size (Minamoto *et al*, 1994b).

Genetic alterations in the adenoma–carcinoma sequence comprise two groups (Kinzler and Vogelstein, 1996). The major group is characterised by a mechanism associated with loss of heterozygosity (LOH), which accounts for a significant proportion of tumour suppressor gene (adenomatous polyposis coli (*APC*) or *p53*) inactivation (Baker *et al*, 1989; Powell *et al*, 1992). Additionally, mutational activation of *KRAS* has been found in about 40% of colorectal neoplasias (Bos *et al*, 1987; Forrester *et al*, 1987). This group accounts for at least two thirds of all CRCs. Another group of cancers exhibits a high frequency of DNA microsatellite instability (MSI) caused by inactivation of DNA mismatch repair (MMR) genes such as *hMLH1* (Bronner *et al*, 1994). Inactivation of this gene, which resulted from bialleleic hypermethylation of the promoter, leads to destabilisation of simple DNA repeat sequence in colorectal tumours (Cunningham *et al*, 1998; Herman *et al*, 1998). Tumour suppressor gene inactivation occurs as a consequence of the state of microsatellite instability.

Some molecular differences between FDNs and PNs have been reported. Mutational activation of *KRAS* is a rare event in FDNs, compared with PNs (Fujimori *et al*, 1994; Minamoto *et al*, 1994a; Yamagata *et al*, 1994; Yashiro *et al*, 2001). There are also distinct differences in chromosomal changes between FDNs and PNs (Richter *et al*, 2003). However, no significant difference in incidence has been observed for somatic mutations in *APC* and *p53* (Olschwang *et al*, 1998; van Wyk *et al*, 2000). Thus, molecular analysis of FDNs is important for achieving a better understanding of the mechanism in the development of CRCs.

The Ras/Raf/MEK/mitogen-activated protein kinase (MAPK) (MEK is the MAPK or extracellular signal-related kinase (ERK) kinase) cascade mediates cellular response to growth signals (Peyssonnaux and Eychene, 2001). Somatic mutations of the *Ras* gene, leading to activation of this signalling pathway and malignant transformation, are frequently observed in protruding tumours (Fujimori *et al*, 1994; Minamoto *et al*, 1994a; Yamagata *et al*, 1994; Yashiro *et al*, 2001; Chan *et al*, 2003). Davies *et al* (2002) have reported the presence of *BRAF* mutations in human cancers such as melanomas, colorectal, and ovarian cancers. *BRAF* mutations in these cancers are oncogenic. BRAF proteins, Ras-regulated kinase, phosphorylate MEK1 and MEK2, which in turn phosphorylate MAPK-ERK 1/2. The activated version of *BRAF*, at least in part, promotes cell proliferation by signalling through MEK and ERK (Davies *et al*, 2002). Moreover, *BRAF* mutations occur in a mutually exclusive relationship with *KRAS* mutations, and are closely related to the carcinogenesis of sporadic CRCs with high-frequency MSI (MSI-H) (Rajagopalan *et al*, 2002; Wang *et al*, 2003).

Recently, molecular characteristics of serrated adenomas (SAs) are reported to be different from those of nonserrated neoplasia. *BRAF* mutations are frequently observed in SAs or hyperplastic polyps (Chan *et al*, 2003; Kambara *et al*, 2004; Konishi *et al*, 2004). However, it remains unknown whether *BRAF* mutations or the status of phosphorylated MAPK (p-MAPK) are the contributors to the tumorigenesis of FDNs, a new type of precursors of CRC. Thus, we evaluated these statuses between flat-depressed and protruding nonserrated neoplasia.

The aim of this study was to investigate the incidence of *BRAF* mutations in a sizable number of FDNs and its implication for *KRAS* mutations, for MSI, and for the immunohistochemical status of p-MAPK, and to compare these genetic and immunohistochemical characteristics of FDNs with PNs as controls.

## MATERIALS AND METHODS

### Subjects

A total of 46 FDNs from 44 patients who underwent endoscopic (*N*=33) or surgical resection (*N*=13) at Showa University Hospital between April 1998 and January 2004 were used for this study. A series of 57 endoscopically or surgically resected PNs were used as controls. We excluded patients who had familial adenomatous polyposis, hereditary nonpolyposis colorectal cancers, or hyperplastic polyposis, and patients with sporadic SAs. Specimen collection procedures and genetic analysis were approved by the ethical committee of Showa University School of Medicine.

### Macroscopic criteria

Macroscopically, each neoplastic lesion was classified as an FDN or a PN, according to the modified criteria described previously (Konishi *et al*, 2003). Briefly, FDNs were defined as slightly mucosal elevations with a flat or slightly rounded surface and a height of less than half of the diameter of the lesions, usually consisting of dysplastic mucosal thickness less than twice that of the adjacent nondysplastic mucosa by histology. Flat-depressed neoplasias were subclassified into flat or depressed neoplasias, depending on whether a depressed component was present. Protruding neoplasias were defined as protruding lesions with or without stalks (sessile, semipedunclated, or pedunculated lesions).

### Histological evaluation

Serial sections (3 *μ*m) were cut from paraffin blocks, and prepared for hematoxylin–eosin (HE) staining and immunostaining. All HE-stained sections were reviewed by a single pathologist (YH), who was blinded to the colonoscopic findings. Dysplastic mucosal lesions were classified as adenomas. When tumour cells had spread through the muscularis mucosa into the submucosa, the lesion was defined as a carcinoma. According to the criteria described previously (Konishi *et al*, 1999), tumour location was classified into three groups: rectum, left-colon (left-c), and right-colon (right-c). Other histopathological features were determined according to the general rules of the Japanese Research Society for Cancer of the Colon and Rectum (Japanese Research Society for Cancer of the Colon and Rectum, 1997).

### DNA preparation

To extract genomic DNA, five adjacent sections (5-*μ*m thick) were obtained from an archival block of formalin-fixed, paraffin-embedded tumour tissue for each macroscopic type. One section was stained with HE, and the percentage of tumour cells was estimated microscopically. The extraction of genomic DNA was described previously (Yamamoto *et al*, 2003). If representative tumour samples contained less than 80% tumour cells, separate samples were obtained from the histological slide for tumourous or adjacent normal tissue using laser-capture microdissection, as described previously (Yamamoto *et al*, 2003). DNA samples from normal colonic mucosa (frozen or formalin-fixed tissue) or peripheral blood were used as normal controls for molecular analysis.

### Mutations in BRAF and KRAS

Primers for exons 11 and 15 were used to evaluate BRAF mutations (Davies *et al*, 2002; Chan *et al*, 2003). These primers included the region of mutation ‘hot spots’ previously identified in this gene. PCR amplification of exon of a KRAS-containing codon 12 or 13 was performed using previously described primers (Brose *et al*, 2002). Mutational screening of the BRAF and KRAS genes was performed by direct sequencing methods, as previously reported (Makino *et al*, 2000). The PCR products were separated by electrophoresis on 2% agarose gels and eluted with GenElute™ Minus EtBr Spin Columns (Sigma, Saint Louis, MO, USA). The purified sample was sequenced using an automated sequencer. All mutations were reconfirmed by independent PCR reactions and sequencing.

### Analysis of MSI

PCR was performed to amplify DNA samples from the tumours and the adjacent normal tissues using an established PCR protocol (Konishi *et al*, 2004). Five microsatellite loci analysed in this study were BAT25, BAT26, D2S123, D5S346, and D17S250 (Boland *et al*, 1998). Tumours showing novel peak patterns were evaluated as MSI positive. A single observer (HN) performed the MSI analysis, and positive or equivocal samples were re-evaluated by a second observer (KN). A tumour sample was considered to contain high-frequency MSI (MSI-H) if two or more of the five informative markers demonstrated instability, and was considered to have low-frequency MSI (MSI-L) when only one marker was unstable (Boland *et al*, 1998). All PCR reactions were repeated on the same sample and only consistent changes in the duplicate reactions were scored as abnormalities.

### Immunohistochemical staining and evaluation of p-MAPK

Deparaffinised and rehydrated sections were heated in a microwave oven in sodium citrate buffer (pH 6.0) for 30 min to retrieve antigens. Endogenous peroxidase activity was inhibited by incubation with 3% hydrogen peroxide for 5 min. Sections were incubated overnight with polyclonal anti-phospho-p44/42 MAPK antibody (Anti-ACTIVE MAPK pAb; Promega, Madison, WI, USA) at 4°C. This specifically recognises the dually phosphorylated, active form of MAPK (p44/ERK1 and p42/ERK2). The working dilution was 1 : 300. Sections were then incubated with horseradish peroxidase-binding amino-acid polymer for 30 min (Histofine Simplestain MAX-PO kit, Nichirei, Tokyo, Japan). Colour was developed by staining with a diaminobenzidine solution. Sections were lightly counterstained with haematoxylin.

**Figure 1:**
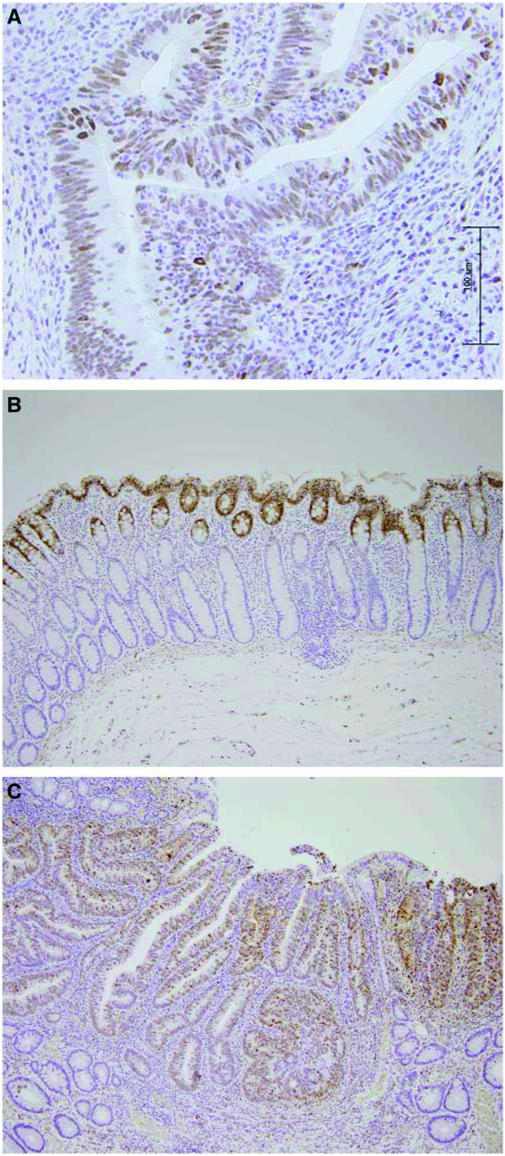
Immunohistochemical staining of phosphorylated mitogen-activated protein kinase (p-MAPK). (**A**) Nuclear expression in a colonic neoplasia. (**B**) In type A, p-MAPK-positive cells were localised within the upper area of the adjacent normal mucosa (× 10). (**C**) In type C, p-MAPK-positive cells were localised in the upper, middle through lower area of the tumour (× 10).

Each immunostained section was examined under a light microscope and evaluated for the nuclear staining (Figure 1A) twice by a senior pathologist (MT) who did not have any knowledge of the clinical and molecular analysis of those samples. At the present, there are no validated criteria for evaluating immunohistochemical staining for p-MAPK; therefore, we used a grading system for evaluating p-MAPK staining based on staining distribution. The dysplastic or normal mucosa glands were divided into three equal areas (upper, middle, and lower). P-MAPK-positive cells were classified into three types (Figure 1): type A, localised within the upper area only; type B, localised in the upper and middle; type C, localised in the upper through lower. We analysed the immunostaining of p-MAPK separately in tumour and adjacent normal tissue. Unfortunately, two paired PNs and adjacent normal mucosa, and five samples of adjacent normal mucosa were not informative for p-MAPK immunostaining, because of the small amounts of tissue in the blocks.

### Statistical analysis

Mann–Whitney *U-*test, *χ*^2^ test, and Fisher's exact test were used for statistical analysis. A value of *P*<0.05 was considered significant.

## RESULTS

There were no significant differences in gender, age, family history of CRC, location, size, or histology between the two macroscopic types. The incidence of accompanying adenoma in the Duke's type A carcinomas was lower in the FDNs than in the PNs (four out of 17 and 12 out of 13, respectively; *P*=0.0002 by Fisher's exact test) (Table 1).

*BRAF* mutations were detected in four out of 46 of FDNs (9%) and none of the 57 PNs. This difference was statistically significant (*P*=0.0369 by Fisher's exact test). Three *BRAF* mutations were found in exon 15 and two were in exon 11 (Table 2). One tumour had *BRAF* mutations in both exons. Two exon 15 mutations observed in depressed neoplasias were the conversion of valine to glutamic acid at codon 599. The remaining findings were novel mutations, P452T (exon 11) in two tumours and T588I (exon 15) in one tumour. Of the four FDNs with *BRAF* mutations, no *BRAF* mutation was detected in the adjacent normal mucosa.

*KRAS* mutations were observed in none of 46 FDNs and 14 out of 57 of PNs (25%). There was significant difference in the incidence of *KRAS* mutations between FDNs and PNs (*P*=0.0002 by Fisher's exact test). All but one *KRAS* mutation was detected in codon 12 of the *KRAS* gene (Table 2).

For the MSI analysis, MSI-H was shown in seven out of 44 FDNs (16%) and in one out of 52 PNs (2%). This incidence of MSI-H differed significantly between FDNs and PNs (*P*=0.022 by Fisher's exact test). MSI-L was shown in 16 out of 44 FDNs (36%) and in six out of 52 PNs (12%: *P*=0.0066 by Fisher's exact test). Of the seven FDNs with MSI-H, four were adenomas and three were Duke's type A carcinomas, whereas one PN with MSI-H were adenomas. *BRAF* mutations were found in one out of seven FDNs with MSI-H (14%) and in three out of 37 FDNs without MSI-H (8%). However, these differences were not statistically significant. No *KRAS* mutation was observed in any PN with MSI-H.

The p-MAPK protein was detected immunohistochemically in all samples to a variable extent. Type B and C immunostaining for p-MAPK was observed in 34 out of 46 FDNs (72%), compared with 24 out of 55 PNs (44%). This difference was statistically significant (*P*=0.0022 by *χ*^2^ test). However, about 80% of the adjacent normal mucosa showed type A p-MAPK expression. There was no significant difference in the incidence of type A p-MAPK expression in the adjacent normal mucosa between FDNs and PNs (34 out of 46 and 42 out of 50, respectively; *P*=0.2241 by *χ*^2^ test).

We compared the clinicopathological and molecular characteristics between neoplasia with type A and with type B/C immunostaining of p-MAPK (Table 3). In the FDNs, type B/C expression of p-MAPK was observed significantly more frequently in Duke's A carcinomas than in the adenomas (*P=*0.0338 by Fisher's exact test). We observed no significant difference in the incidence of type B/C immunostaining of p-MAPK between FDNs with and without *BRAF* mutations. In contrast, type B/C immunostaining for p-MAPK was detected more frequently in large (⩾10 mm) than small (<10 mm) PNs. This size-related difference was statistically significant (*P=*0.0265 by Fisher's exact test). Type B/C immunostaining of p-MAPK was demonstrated more frequently in PNs with *KRAS* mutations than without *KRAS* mutations (*P=*0.0272 by Fisher's exact test).

## DISCUSSION

*BRAF* status has been examined in a variety of human malignancies. *BRAF* mutations have been reported in approximately 10% of CRCs (Davies *et al*, 2002; Rajagopalan *et al*, 2002; Fransen *et al*, 2004). However, the status of the *BRAF* gene in the precursor lesions of CRCs has not been thoroughly explored, and there is morphological heterogeneity in the oncogenesis (Shimoda *et al*, 1989). Flat-depressed and protruding adenomas may be the precursors to cancers arising *de novo* and to polypoid cancers, respectively. To our knowledge, this is the first study of the mutational status of *BRAF* in terms of the morphological characteristics of colorectal nonserrated neoplasias. Protruding neoplasias have a significantly higher frequency of *KRAS* mutation than flat neoplasias, despite the similarity of the tumour size (Fujimori *et al*, 1994; Minamoto *et al*, 1994a; Yamagata *et al*, 1994; Yashiro *et al*, 2001). We identified four FDNs (9%) with mutations in *BRAF*, but no PNs with the mutations. By contrast, *KRAS* mutations were observed in none of FDNs and in 25% of PNs. *BRAF* and *KRAS* mutations were mutually exclusive in the morphologically distinct nonserrated neoplasias (FDNs and PNs, respectively).

The mutational spots of *BRAF* gene cluster within the activation segment (exon 15) and the G-loop (exon 11) of the kinase domain, which are highly conserved among serine/threonine kinases throughout evolution. In our series, all of the mutations in exon 15 of *BRAF* observed in depressed neoplasias (DNs) involved conversion of valine to glutamic acid at codon 599 (V599E). This V599E mutation is a ‘hot spot’ mutation of colorectal cancers, as well as other human cancers (Davies *et al*, 2002; Rajagopalan *et al*, 2002; Wang *et al*, 2003; Yuen *et al*, 2003; Domingo *et al*, 2004; Fransen *et al*, 2004; Koinuma *et al*, 2004). Although we need more extensive analysis, V599E mutations might contribute to tumorigenesis in DNs. The other non-V559 mutations detected here were the novel mutations, P452T (exon 11) and T588I (exon 15), in CRCs. This T588I mutation may be associated with increased MAPK activity because the tumour with this mutation showed a type B and C expression of p-MAPK protein.

Continuous activation of the MAPK signalling pathway is of critical importance for the development of CRCs. P-MAPK forms (phosphorylated ERK1 and ERK2) translocate to the nucleus to modulate gene expression through the activation of transcriptional factors (Peyssonnaux and Eychene, 2001). Nuclear staining was observed here as a positive reaction for p-MAPK protein, and type B and C immunostaining of p-MAPK was frequently observed in the FDNs or PNs. The incidence of type B and C immunostaining of p-MAPK was significantly higher in the FDNs than in the PNs. Thus, abnormal accumulation of p-MAPK protein is more likely to be associated with the tumorigenesis of FDNs than of PNs.

Mutational activation of *BRAF* or *KRAS* gene signals act through the classical MAPK cascade to promote proliferation (Davies *et al*, 2002). We found that type B/C expression of p-MAPK was more frequently observed in PNs with a *KRAS* mutation. Therefore, the mutational type of *KRAS* might activate the MAPK pathway more strongly than the wild-type *KRAS* (Vojtek and Der, 1998). Type B/C immunostaining of p-MAPK was observed more frequently in the large PNs than in small PNs. The *KRAS* gene mutation frequency in colorectal polyps increases in proportion to their size (Vogelstein *et al*, 1988). Of the 14 PNs with *KRAS* mutations in this series, 11 (79%) were adenomas. Thus, derangement of the MAPK signalling pathway may be an early, size-dependent event in the tumorigenesis of PNs, and correlates to the status of *KRAS* mutation. On the other hands, there was no significant difference in the incidence of type B/C immunostaining of p-MAPK between FDNs with and without *BRAF* mutations. Type B/C immunostaining of p-MAPK was more common in Duke's A carcinomas than in adenomas of FDNs. This implies that abnormal accumulation of p-MAPK protein may be a critical event in the tumour progression of FDNs, independently of *BRAF* mutations.

Our immunohistochemical data suggest that alterations of the MAPK pathway are important for the development of FDNs, but may also highlight new therapeutic strategies for dealing with CRCs that arise from FDNs. As more than 70% of FDNs show positive immunostaining of p-MAPK, this signalling pathway may play an important role in the tumorigenesis of FDNs. Many have reported CRCs arising from FDNs or nonpolypoid neoplasias (Shimoda *et al*, 1989; Bedenne *et al*, 1992; Iishi *et al*, 1992; Kudo, 1993; Minamoto *et al*, 1994b; Konishi *et al*, 1999; Rembacken *et al*, 2000; Kaneko *et al*, 2004). Therefore, inhibition of either p-MAPK or related molecule might be a new therapeutic strategy to treat these CRCs. CI-1040 (PD184352) is highly selective inhibitor of the MAPK signalling cascade specifically targeting the inhibition of MEK (Delaney *et al*, 2002). Antitumour activity was found to correlate with CI-1040-mediated inhibition of phosphorylated ERK levels. However, *BRAF* mutations are infrequent in FDNs. Moreover, no significant mutations in *ARAF* or *RAF-1* have been found in CRCs (Fransen *et al*, 2004). However, the Raf-MEK-ERK pathway is regulated via the interaction with and modulation of the function of a wide range of signalling proteins (English *et al*, 1999; Zimmermann and Moelling, 1999; Kolch, 2000). Therefore, further investigation is required to clarify what leads to the tumour-specific expression of p-MAPK in FDNs.

We found MSI-H in seven of 44 FDNs (16%) but in only one of 52 PNs (2%). Of the seven FDNs with MSI-H, four were adenomas and three were Duke's A carcinomas. Olschwang *et al* (1998) reported that eight out of 36 flat colorectal neoplasias showed MSI-H (22%). The frequency of MSI-H in that series did not differ with regard to the histological type. However, Yashiro *et al* (2001) observed no significant difference in the incidence of MSI-H between flat-type and polypoid-type cancers (four out of 25 and zero out of 25, respectively). Selection of tumour samples may explain this difference. In our series, 41% of FDNs showed depressed type morphology and about two-thirds of FDNs were 10 mm or more in diameter. However, they also suggested that some flat neoplasias may progress to *de novo* cancers with LOH at chromosome 3p, the location of *hMLH1*, and this could explain the onset of MSI-H as an alternative mechanism to hypermethylation of the promoter of *hMLH1*. Previous studies (Rajagopalan *et al*, 2002; Wang *et al*, 2003; Domingo *et al*, 2004; Koinuma *et al*, 2004) have reported that *BRAF* mutations occur more frequently in microsatellite-unstable than in microsatellite-stable CRCs. However, we observed no significant difference in the incidence of *BRAF* mutations between FDNs with and without MSI-H.

In summary, *BRAF* and *KRAS* mutations were mutually exclusive in the morphological characteristics of colorectal nonserrated neoplasias. High-frequency microsatellite instability was significantly more frequently seen in FDNs than in PNs. Therefore, it is possible that some FDNs with *BRAF* mutation or MSI-H progress to *de novo* type cancers (ie, flat or depressed cancers without accompanying adenoma). Abnormal accumulation of p-MAPK protein seems to be more frequently implicated in the tumorigenesis of FDNs than that of PNs. However, this accumulation was significantly correlated with the incidence of *KRAS* mutations in PNs, but not to that of *BRAF* mutations in FDNs. Derangement of the MAPK pathway in FDNs might occur via the genetic alteration other than *BRAF* or *KRAS* mutation.

## Figures and Tables

**Table 1:** Clinicopathological characteristics of patients with flat and depressed neoplasias, and protruding neoplasia

	**FDNs (*N*=46)**	**PNs (*N*=57)**	***P*-value**
*Gender*			
Male/female	34/10	34/16	0.3159*
Mean age (year)	66.1	65.4	0.7001**
(Range)	(41–85)	(32–82)	
			
*Family history of CRC*			
Present	4	3	0.3158*
Absent	35	45	
Unknown	5	2	
			
*Location*			
Left-c and rectum	17	26	0.3575*
Right-c	29	31	
			
*Size*			
<10 mm	14	23	0.2970*
⩾10 mm	32	34	
			
*Macroscopic type*			
Flat	27	NA	
Depressed	19		
			
*Histology*			
Adenoma	29	44	0.1161*
Dukes' A carcinoma	17	13	
			
*Accompanying adenoma in Dukes' A carcinoma*			
Present	4	12	0.0002***
Absent	13	1	

FDNs=flat and depressed neoplasias; PNs=protruding neoplasias; CRC=colorectal cancers; NA=not applicable.

* *P*-value calculated by *χ*^2^ test;

** *P*-value calculated by Mann–Whitney *U*-test;

*** *P*-value calculated by Fisher's exact test.

**Table 2:** Characteristics of colorectal neoplasias with *BRAF* or *KRAS* mutations

**Sample**	**Location**	**Size (mm)**	**Histology**	**Sequence change**		**Codon**	**Amino-acid substitution**	**p-MAPK^a^**
*BRAF mutations*								
DN	A	25	Dukes' A	1796	T → A	599	V → E	B
FN	D	10	Adenoma	1354	C → A	452	P → T	A
FN	T	11	Adenoma	1763	C → T	588	T → I	C
DN	T	8	Adenoma	1354	C → A	452	P → T	A
				1796	T → A	599	V → E	
								
*KRAS mutations*								
PN	A	12	Adenoma	35	G → T	12	G → V	B
PN	A	3	Adenoma	35	G → C	12	G → A	A
PN	D	9	Adenoma	35	G → A	12	G → D	B
PN	S	8	Adenoma	35	G → C	12	G → A	C
PN	A	32	Adenoma	35	G → T	12	G → V	B
PN	S	35	Adenoma	35	G → A	12	G → D	A
PN	A	50	Adenoma	35	G → T	12	G → V	B
PN	C	40	Dukes' A	34	G → T	12	G → C	A
PN	R	30	Dukes' A	35	G → A	12	G → D	B
PN	A	7	Adenoma	35	G → C	12	G → A	B
PN	T	40	Dukes' A	35	G → A	12	G → D	A
PN	T	12	Adenoma	35	G → A	12	G → D	B
PN	T	30	Adenoma	35	G → A	12	G → D	B
PN	R	35	Adenoma	38	G → A	13	G → D	B

a Evaluating systems for immunohistochemical staining for p-MAPK are described in Materials and Methods. P=positive immunostaining; N=negative immunostaining; FN=flat neoplasia; DN=depressed neoplasia; PN=protruding neoplasia; R=rectum; S=sigmoid; D=descending; T=transverse; A=ascending colon; C=cecum; Dukes' A=Dukes' A carcinoma; p-MAPK=phosporylated mitogen-activated protein kinase.

**Table 3:** Expression of phosphorylated MAPK in flat-depressed and protruding neoplasias compared with clinicopathological and molecular characteristics

	**Expression of p-MAPK**					
	**FDNs (*N*=46)**			**PNs (*N*=55)**		
	**Type A**	**Type B/C**	***P*-value**	**Type A**	**Type B/C**	***P*-value**
*Location*						
Left-c and rectum	3	14	0.4893*	13	12	0.5514**
Right-c	9	20		18	12	
						
*Size*						
<10 mm	3	11	0.7294*	16	5	0.0265*
⩾10 mm	9	23		15	19	
						
*Macroscopic type*						
Flat	9	18	0.3071*	NA		
Depressed	3	16				
						
*Histology*						
Adenoma	11	18	0.0338*	23	19	0.7561*
Dukes' A carcinoma	1	16		8	5	
						
*BRAF mutation*						
Mut+	2	2	0.2758*	0	0	NA
Mut−	10	32		31	24	
						
*KRAS mutation*						
Mut+	0	0	NA	4	10	0.0272*
Mut−	12	34		27	14	
						
*MSI* a						
MSS/MSI-L	11	26	0.6532*	30	19	>0.999*
MSI-H	1	6		1	0	

Evaluating systems for immunohistochemical staining for p-MAPK are described in Materials and Methods.

a The numbers for MSI reflect the numbers of cases among the informative cases.

p-MAPK=phosphorylated mitogen-activated protein kinase; FDNs=flat and depressed neoplasias; PNs=protruding neoplasias; Left-c=sigmoid and descending colon; Right-c=transverse, ascending colon, and cecum; Mut+=presence of mutation; Mut−=absence of mutation; MSI=microsatellite instability; MSS=microsatellite stable; MSI-L=low-frequency MSI; MSI-H=high-frequency MSI; NA=not applicable.

* *P*-value calculated by Fisher's exact test;

** *P*-value calculated by *χ*^2^ test.
